# Clinical and demographic factors associated with change and maintenance of disease severity in a large registry of patients with rheumatoid arthritis

**DOI:** 10.1186/s13075-017-1289-x

**Published:** 2017-04-27

**Authors:** George W. Reed, David H. Collier, Andrew S. Koenig, Katherine C. Saunders, Dimitrios A. Pappas, Heather J. Litman, Joel M. Kremer, Sameer Kotak

**Affiliations:** 1 University of Massachusetts Medical School, 55 Lake Ave North, Worcester, MA 01605 USA; 2 Amgen Inc., Thousand Oaks, CA USA; 3 Pfizer Inc., Collegeville, PA USA; 4 Corrona LLC, Southborough, MA USA; 5 Columbia University College of Physicians and Surgeons, New York, NY USA; 6 Albany Medical College and The Center for Rheumatology, Albany, NY USA

**Keywords:** Rheumatoid arthritis, Disease activity, Prediction, Markov model, Clinical Disease Activity Index

## Abstract

**Background:**

We examined models to predict disease activity transitions from moderate to low or severe and associated factors in patients with rheumatoid arthritis (RA).

**Methods:**

Data from RA patients enrolled in the Corrona registry (October 2001 to August 2014) were analyzed. Clinical Disease Activity Index (CDAI) definitions were used for low (≤10), moderate (>10 and ≤22), and severe (>22) disease activity states. A Markov model for repeated measures allowing for covariate dependence was used to model transitions between three (low, moderate, severe) states and estimate population transition probabilities. Mean sojourn times were calculated to compare length of time in particular states. Logistic regression models were used to examine impacts of covariates (time between visits, chronological year, disease duration, age) on disease states.

**Results:**

Data from 29,853 patients (251,375 visits) and a sub-cohort of 9812 patients (46,534 visits) with regular visits (every 3–9 months) were analyzed. The probability of moving from moderate to low or severe disease by next visit was 47% and 18%, respectively. Patients stayed in moderate disease for mean 4.25 months (95% confidence interval: 4.18–4.32). Transition probabilities showed 20% of patients with low disease activity moved to moderate or severe disease within 6 months; >35% of patients with moderate disease remained in moderate disease after 6 months. Results were similar for the regular-visit sub-cohort. Significant interactions with prior disease state were seen with chronological year and disease duration.

**Conclusion:**

A substantial proportion of patients remain in moderate disease, emphasizing the need for treat-to-target strategies for RA patients.

**Electronic supplementary material:**

The online version of this article (doi:10.1186/s13075-017-1289-x) contains supplementary material, which is available to authorized users.

## Background

Rheumatoid arthritis (RA) is a chronic, systemic inflammatory disease, which can lead to progressive joint destruction and accumulating disability from autoimmune processes that target the synovium [1]. Several effective and safe medications can decrease the level of inflammation and achieve a low disease activity state or even remission [2]. Treat-to-target principles emphasize the need for achievement of remission or low disease activity [3]. However, reaching a state of continuous and stable remission or low disease activity is not observed in all patients despite aggressive therapy [4–7], and a number of studies have shown that moderate or high disease activity is not uncommon in treated patients [8–12]. RA disease activity in treated patients frequently fluctuates over time. Continuous surveillance by the treating physician is therefore needed to maintain disease activity at low levels and diminish the probability of joint damage and associated disability. Medications may fail to control the disease activity for long periods of time because of development of resistance, the autoimmune process maintaining the RA-related inflammation may become more active, and the disease activity may flare.

The current therapy guidelines provide a therapy algorithm, which recommends all patients should be treated with conventional synthetic disease-modifying antirheumatic drugs (csDMARDs) as first-line therapy, or if they fail to achieve low disease activity or remission with a tumor necrosis factor inhibitor (TNFi). If there is primary or secondary treatment failure and the patient returns to a state of moderate disease activity, a different TNFi can be tried, followed by switch to a non-TNFi biologic DMARD (bDMARD) after failure of a second TNFi [3]. However, this model of therapy does not take into account characteristics that may affect the probability of a patient to return to low disease activity or remission regardless of therapy. It is possible that the previous history of disease activity fluctuation, coupled with other patient and disease characteristics, may be able to predict the future “behavior” of the disease and thus decisions about the aggressiveness of therapy could be tailored according to these probabilities.

For patients with moderate disease activity, the evolution of disease activity over time is particularly difficult to predict currently. Understanding the patterns of fluctuation as patients transition out of moderate disease activity provides an assessment of the unmet needs in the RA patient population regardless of treatment or duration of disease.

The most common modeling techniques that have been used to predict evolution of disease activity in RA include decision trees, Markov models, individual sampling methods, and discrete event simulation [13]. These have mainly been applied to perform cost effectiveness and cost utility analyses using real or simulated populations [13–16]. Few studies have used the models to examine clinical trends in RA development and factors associated with these transitions. In the current study we examined the feasibility of using Markov models and clinical registry data to estimate transition probabilities and the impact of covariates on these transitions. We focused on the group of patients with moderate disease activity and compared mean times that these patients remained in their current disease state to patients in other disease states. We used the Corrona registry database as the clinical patient population to provide a proof of principle that these models can potentially be used to evaluate clinically relevant associations with disease state transitions.

## Methods

### Patients

Patients evaluated in the current analyses were enrolled in the Corrona registry. The Corrona registry has been described in detail elsewhere [17]. Briefly, the Corrona registry comprises a prospective US observational cohort of patients with arthritis who are enrolled by participating rheumatologists [17, 18]. The Corrona registry was founded in 2001. The Corrona Rheumatology Practice Network is composed of more than 100 private and academic practices across 42 states within the USA, with more than 350 rheumatologists contributing data. All geographic regions in the continental USA are represented and there are no restrictions on age, disease activity, or disease duration or other restrictions to patient participation in the registry.

Data are collected from both patients and their treating rheumatologists, who gather information on disease duration, prognosis, disease severity and activity, medical comorbidities, use of medications including csDMARDs and bDMARDs, and adverse events. Follow-up assessments are requested at least as often as every 6 months and completed during routine clinical encounters. At each Corrona registry visit patients and physicians record data on disease severity and activity, RA and other medications, adverse events, quality of life, selected laboratory and imaging results, and sociodemographic information. Visits have occurred at a median interval of 4.4 months.

The study population used for analysis of the impact of intervals between visits consisted of 29,853 RA patients with 251,375 visits with Clinical Disease Activity Index (CDAI) components measured at all visits in Corrona from October 2001 to August 2014. Only 1.2% of patients (n = 371) were dropped because of a visit with no CDAI assessment. A subset of patients with all visit intervals between 3 and 9 months (regular-visit sub-cohort) was used for covariate analyses beyond visit length, and included 9812 RA patients with 46,534 visits.

### Measurement of disease activity

Disease activity was measured using the CDAI, an established composite measure of disease activity [19]. CDAI is calculated based on the number of swollen and tender joints, the patient’s self assessment of overall RA disease activity (scale of 1–10), and the physician’s (or evaluator’s) assessment of overall RA disease activity (scale of 1–10 where 10 is maximal activity). CDAI uses the 28-joint scale to evaluate the number of swollen and tender joints - thus ankles and feet are excluded. Inflammation markers are not included in CDAI calculation. CDAI cutoffs have been established to define different disease activity states; remission is defined by a CDAI ≤2.8, low disease activity is defined by a CDAI >2.8 and ≤10, moderate disease activity is defined by a CDAI >10 and ≤22 and high disease activity is defined by a CDAI >22 [19]. A patient with moderate disease activity was identified when the patient’s CDAI score changed from low disease activity or remission to moderate disease activity. This patient was then followed for any disease activity transitions from that initial transition to a moderate state.

### Model disease activity states

#### Model

RA patients are considered to transition over time among disease states defined by CDAI. While the disease activity score is a continuous scale, the discrete states are used for clinical guidance. Markov models provide a convenient probability model for examining discrete states and estimating the likelihood of patient transitions among states. They can be estimated with simple models based on a patient’s current state or more complex models that account for patient transition history. The models provide population-based estimates on transitions and with the inclusion of patient covariates can and would provide clinically relevant information.

**Fig. 1:**
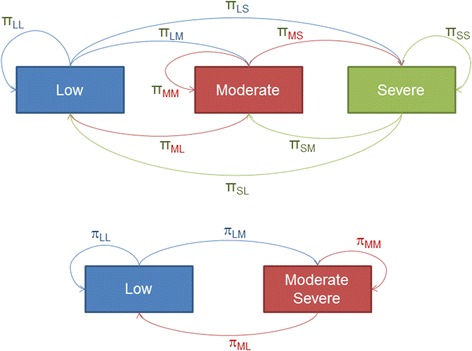
Transition probabilities for the 3-state model of disease severity. *π* probability, *LL* transition from low to low activity, *LS* transition from low to severe activity, *LM* transition from low to moderate activity, *MM* transition from moderate to moderate activity, *ML* transition from moderate to low activity, *MS* transition from moderate to severe activity, *SM* transition from severe to moderate activity, *SL* transition from severe to low activity, *SS* transition from severe to severe activity

Figure 1 illustrates a model using three states (remission/low, moderate, and high) where transition probabilities (π_ij_) are defined as the probability of moving from one state to another (i ≠ j), or remaining in the same state (i = j). For example, π_LM_ represents the probability of transitioning from low disease to moderate disease while π_LL_ represents the probability of remaining in low disease state.

A full description of the Markov chain methodology is provided in the Additional file 1.

#### Model fit and variable time intervals

Within the model framework the impact of a range of covariates on patient movement among disease states can be examined. With clinical registry data collected at a patient’s usual clinical visit, the time between visits will vary and it is important to first assess the potential confounding of the time between visits on transition probabilities to understand adjustments to the model that may be needed for visit interval variation.

The 3-state model was fit using multinomial logistic regression models with a robust variance estimator to account for remaining intragroup (within patient) correlation across visits. The 3-state model was also fit using a multi-state model to describe how an individual moves between states and fitting the transition probability matrix over an interval of 6 months. Mean months to switching states were estimated based on the probabilities of the next move of the process [20]. The current disease activity state was modeled as a function of the prior state and the time between visits divided into quintiles. The interaction of prior state and time between visits was included in the model to examine the impact of time interval on estimated transition probabilities. In addition, predicted transition probabilities from the multinomial model were estimated for the full population and for those patients with all visit intervals between 3 and 9 months (regular-visit sub-cohort) to examine the impact of variability of visits on the model estimates.

Additional fits used the 2-state model to illustrate the model fitting and interpretation. A logistic regression model for the current disease state was fitted with a robust variance estimator. The interaction of select covariates and the prior state were used in the model. We examined three factors (in addition to controlling for visit interval): year (to examine trends over time based on evolving treatment guidelines and availability of new treatments), disease duration (stability of disease with duration), and age (impact of aging on disease variability). Covariates were measured at each time point (visit). All models were adjusted for the length of time between visits (3–4, 4–5, 5–6, 6–7, 7–8, 8–9 months). Covariates examined were chronological year (2001–2004, 2005–2006, 2007–2008, 2009–2010, 2011–2012, 2013–2014), duration of disease (0–3, 3–10, >10 years) and age (<50, 50–64, ≥65 years).

## Results

Patient demographic and clinical characteristics are shown in Table 1. The regular-visit sub-cohort was similar to the full population except for slightly lower disease activity, functionality, and duration of RA. The interquartile range (IQR) for visit intervals in the full population was 3.5–6.2 months (mean 5.4 months; standard deviation (SD) 3.1). In the regular-visit sub-cohort the IQR was 4.0–6.2 months (mean 5.3 months; SD 1.5). There were 8304 patients among the full population that were in moderate disease activity at their first visit as defined in “Methods.”

**Table 1:** Patient demographic and clinical characteristics

	Full population (excluding those with regular visits) (n = 20,041)	Regular visits sub-cohort (n = 9812)
Age, mean years (SD)	57.9 (13.4)	59.3 (13.5)
Sex, *n* female (%)	15,193 (76.3)	7360 (75.8)
Duration of RA, mean years (SD)	9.1 (9.7)	8.7 (9.7)
Disabled, *n* (%)	2439 (12.3)	1072 (11.1)
Patient pain^a^, mean score (SD)	34.6 (27.7)	33.6 (27.8)
TJC28, mean *n* (SD)	4.6 (6.2)	4.1 (5.8)
SJC28, mean *n* (SD)	4.5 (5.6)	3.6 (5.0)
PtGA, mean score (SD)	32.0 (26.7)	30.9 (26.6)
PGA, mean score (SD)	25.7 (21.9)	23.5 (21.5)
CDAI, mean score (SD)	14.8 (13.3)	13.0 (12.6)
CDAI category, *n* (%)		
Low (score >2.8 to ≤10)	9435 (47.1)	5269 (53.7)
Moderate (score >10 to ≤22)	5674 (28.3)	2634 (26.8)
Severe (score >22)	4929 (24.6)	1909 (19.5)
History of biologic DMARD use, *n* (%)	9411 (47.0)	4485 (45.7)

*SD* standard deviation, *RA* rheumatoid arthritis, *TJC28* tender joint count based on 28 joints, *SJC28* swollen joint count based on 28 joints, *PtGA* patient global assessment, *PGA* physician global assessment, *CDAI* Clinical Disease Activity Index, *DMARD* disease-modifying antirheumatic drug

^a^Patient pain was rated on a visual analog scale of 0–100

Table 2 shows the estimated transition probabilities using the full population from the 3-state model based on the multinomial model. Probabilities using the regular-visit cohort were similar, with differences in the estimates ranging from 0.0 to 0.05 for the 3-state model. Table 2 also presents the estimated transition probabilities from the 3-state model considering a multi-state model over an interval of 6 months; these estimates are similar to the original 3-state model. Among patients in moderate disease activity, mean time to another state was 4.25 months (standard error (SE) 0.04 months), meaning that the mean time that a patient remained in moderate disease activity was 4.25 months. This estimate was greatly increased among patients in low disease activity (mean 19.38 (SE 0.15) months) and similar among patients in severe disease activity (mean 5.30 (SE 0.06) months).

**Table 2:** Estimated transition probabilities

	Prior state	Current state		
		Low	Moderate	Severe
3-State model				
Full population	Low	0.82	0.15	0.03
	Moderate	0.47	0.35	0.18
	Severe	0.13	0.39	0.48
Regular-visit sub-cohort	Low	0.84	0.13	0.03
	Moderate	0.52	0.33	0.15
	Severe	0.16	0.41	0.43
3-State model assuming a 6-month interval				
Full population	Low	0.81	0.15	0.05
	Moderate	0.46	0.37	0.17
	Severe	0.26	0.33	0.41
Regular-visit sub-cohort	Low	0.84	0.13	0.03
	Moderate	0.48	0.38	0.14
	Severe	0.28	0.34	0.38

We further assessed the impact of the length of visit intervals on transition probabilities using the interaction of visit interval and prior state in the regular-visit sub-cohort. Additional file 1: Tables S1 and S2 show the estimated odds ratios (ORs) of the estimated model with interaction terms and the estimated increased (or decreased) risk of moderate/severe disease if the prior state was low or moderate/severe. A large OR (12.4) suggested an increased risk of the current state being moderate/severe if the prior state was moderate/severe vs low for the reference visit interval of 3–4 months.

The estimated ORs for the risk of transitioning from low disease to moderate disease were all >1, indicating higher odds of transitioning from a prior state of low to a current state of moderate/severe if the visit interval was longer but not all were significant. The estimated ORs for the risk of transitioning from moderate disease to moderate disease were all < 1, indicating lower odds of remaining in moderate disease with longer intervals. Again, the estimated ORs were close to 1 and not all statistically significant. The impact of the length of visit intervals was not large, but suggested that models should be adjusted for this potential confounder.

To illustrate how the model can examine covariate associations with transition probabilities we examined three factors: year, disease duration, and age. Separate models adjusted only for visit intervals (not shown) estimated a significant interaction of the prior disease state and year and disease duration but no significant interaction with age. Table 3 shows the results from the multivariable logistic model using all four factors.

**Table 3:** Covariate associations with transition probabilities

Covariate	OR (95% CI)
Year (reference: 2001–2004)	
Effect on transition from low to moderate/severe disease	
2005–2006	0.865 (0.700 to 1.068)
2007–2008	0.593 (0.471 to 0.746)
2009–2010	0.583 (0.474 to 0.716)
2011–2012	0.615 (0.506 to 0.748)
2013–2014	0.605 (0.497 to 0.735)
Effect on transition from moderate/severe to moderate/severe disease	
2005–2006	1.055 (0.853 to 1.304)
2007–2008	0.911 (0.733 to 1.133)
2009–2010	0.824 (0.679 to 0.999)
2011–2012	0.888 (0.740 to 1.066)
2013–2014	1.004 (0.837 to 1.205)
Duration of RA (reference: 0 to 3 years)	
Effect on transition from low to moderate/severe disease	
3 to 10 years	0.777 (0.698 to 0.865)
>10 years	0.840 (0.752 to 0.939)
Effect on transition from moderate/severe to moderate/severe disease	
3 to 10 years	1.167 (1.045 to 1.303)
>10 years	1.379 (1.236 to 1.539)
Age (reference: <50 years)^a^	
50 to 64 years	1.176 (1.082 to 1.279)
≥65 years	1.024 (0.939 to 1.117)
Visit interval (reference: 3 to < 4 months)	
Effect on transition from low to moderate/severe disease	
4 to <5 months	1.057 (0.940 to 1.187)
5 to <6 months	1.085 (0.961 to 1.226)
6 to <7 months	1.201 (1.057 to 1.364)
7 to <8 months	1.349 (1.160 to 1.568)
8 to 9 months	1.171 (0.977 to 1.403)
Effect on transition from moderate/severe to moderate/severe disease	
4 to <5 months	0.931 (0.831 to 1.042)
5 to <6 months	0.801 (0.712 to 0.902)
6 to <7 months	0.779 (0.686 to 0.886)
7 to <8 months	0.799 (0.683 to 0.935)
8 to 9 months	0.866 (0.729 to 1.028)

*CI* confidence interval, *RA* rheumatoid arthritis

^a^No interaction with prior state and age was noted so the effect was the same for the transition from low to moderate/severe and from moderate/severe to moderate/severe disease

Effects over time were seen in the probability of transitioning from a low disease state to moderate/severe disease with ORs <1 for 2007 onward, indicating a lower likelihood of moving to moderate/severe disease from a low disease state. There was little to no impact on the transition probabilities of remaining in moderate disease with ORs close to 1. Longer disease duration had lower odds for transition from low to moderate/severe disease but higher odds for remaining in moderate/severe disease. Age effects were similar regardless of prior disease state (interaction not significant) so were added to the model only as a main effect. ORs were close to 1, with the OR for age 50–64 years estimated at 1.2, indicating this age group was at a slightly higher risk of ending in moderate/severe disease (whether transitioning from low disease or remaining in moderate/severe disease).

## Discussion

The severity of RA symptoms is currently unpredictable with clinical tools or with laboratory or imaging biomarkers, making management of this chronic disease a challenge for both patients and physicians [21]. Only a fraction of patients will reach constant and longstanding remission while on one or more therapies. More frequently, disease activity fluctuates over time despite therapy, due to a variety of reasons, such as development of resistance to medications, increases in the activity of the RA-related autoimmune process, or other factors that may interfere with medication effectiveness. Several studies of separate populations have shown that a considerable fraction of patients with RA have moderate or high disease activity [8–10]. In our study of a subset of biologic-agent-naïve patients in the Corrona database with disease duration >1 year, rates of low, moderate, and high disease activity were approximately 58%, 30%, and 12%, respectively [22]. The Markov models used in these analyses provide insight into patients with moderate disease and estimated transitions among disease levels in the real world, and provide a proof of concept of using these models to understand covariates associated with transition probabilities even when clinic visits are not at fixed intervals.

Markov models based on real-world data from patients with RA were used in this study to estimate disease state transition probabilities from clinical visit to clinical visit. We analyzed data from all RA patients in the Corrona database and the subset of patients with relatively regular visits with a range of intervals from 3 to 9 months. The transition probabilities remained stable even with longer times between visits, demonstrating the robustness of this methodology when applied to data from real-world practice.

Our results showed that for a patient in low disease activity, the probability of moving to moderate disease was 15% and to severe disease was 3% over an interval of 6 months. Thus over a 6-month period a patient has a 20% chance of moving out of low disease activity. In patients with moderate disease, while there was a 47% probability of moving to low disease, there was still 53% probability of remaining in moderate disease or even moving to severe disease. Patients with severe disease had 48% probability of remaining in the severe state, 39% probability of moving to moderate disease, and only 13% probability of achieving low disease activity. Patients in moderate disease activity remained in that state for an average of only 4 months, whereas patients in low disease activity remain there for an average of approximately 19 months. This illustrates the need to better understand the treatment of patients in moderate disease activity so that their disease can be managed so as to increase the likelihood that a patient will proceed to low rather than high disease activity. A renewed emphasis on the treat-to-target strategy and its timing specifically in patients with moderate disease might be considered.

Modeling approaches like the one used in this study may help us better understand the natural history of the disease in the era of biologic and targeted synthetic medications. Such approaches may be used to evaluate the effectiveness of therapies on a population level, and perhaps identify areas for improvement in therapy and the need for more aggressive treatment. These models indicate an unmet need for an expanded array of treatment options for patients with moderate or severe disease. Notably, these models and observations could have only been obtained from very large data sets, such as the Corrona registry.

The models were able to illustrate changing trends in disease state movement across the years and as duration of disease increased in patients. The lower odds in the later years for transitioning from low to moderate disease illustrate how treatment strategies have developed over time that have enabled patients to remain in low disease activity. However, the impact has not extended to moving at least some patients from moderate to low disease as illustrated by the similar time to transition over the years, another indication of unmet need in the RA population. Longer duration of disease showed lower odds of transitioning from low to moderate disease, which may be an indication of more disease fluctuations at disease onset as best treatments are determined. However, there were greater odds of remaining in moderate disease with longer duration, which may also indicate stability of RA severity in patients with long-standing disease and more difficulty in changing the disease state.

Prior use of Markov models in RA [13–16] have concentrated on health economic models. The proposed models reported here were used as a tool to assess factors that impact the transition probabilities, which would assist in understanding population trends and eventually individual modifiable factors. Markov models assume that a patient is always in one of a finite number of discrete health states, called Markov states. All events are represented as transitions from one state to another. Disease activity is a continuous measure and the discrete Markov states are therefore artifacts that are used to summarize changes in disease activity. However, the spectrum of RA disease severity fits within a clinical paradigm related to treatment changes and “treat-to-target” goals, and provides a simplified model of patient disease fluctuations.

Limitations to the study include restriction to the single index of CDAI (potentially inflated by chronic damage in patients with longstanding disease) and the limited covariates used in proof-of-concept work. Additional disease measures and states and additional covariates can be examined in future work.

## Conclusion

In summary, this work provides a proof of concept of the Markov model for assessing associations with disease state transitions in patients with RA and insights into how treatment trends over time have impacted these transition times. Even with variable visit intervals the model provided robust estimates, and further studies to examine effects of other variables on changes in the severity of RA disease activity are warranted. In addition, our results indicate that while the mean time in moderate disease is short, indicating effective treatment in many patients, there is still a substantial proportion that remain in moderate disease after 6 months (or worse, have an increase in severity to severe disease), indicating an unmet need in the treatment of patients with RA. Future research should aim at determining specific modifiable covariates that can impact disease transitions and allow development of algorithms that could possibly be applied to individual patients to predict fluctuations of disease activity and assist in decisions regarding the aggressiveness of therapy.

## Additional file

Additional file 1: Supplemental methods - Markov chain. **Table S1.** Model with visit interval interaction. **Table S2.** Impact of visit interval on transition probabilities. (PDF 25 kb)

## Abbreviations

bDMARD: Biologic Disease-modifying antirheumatic drug
CDAI: Clinical Disease Activity Index
DMARD: Disease-modifying antirheumatic drug
OR: Odds ratio
RA: Rheumatoid arthritis
SD: Standard deviation
SE: Standard error
