# Supplementary material for: Clinical and demographic factors associated with change and maintenance of disease severity in a large registry of patients with rheumatoid arthritis
Source: Arthritis Res Ther. 2017 Apr 27;19:81. doi: 10.1186/s13075-017-1289-x (PMC5406915; doi:10.1186/s13075-017-1289-x)
Supplement: Additional file 1: — Supplemental methods - Markov chain. Table S1. Model with visit interval interaction. Table S2. Impact of visit interval on transition probabilities. (PDF 25 kb) [file 13075_2017_1289_MOESM1_ESM.pdf]

Clinical and demographic factors associated with change and maintenance of disease severity in a large registry of patients with rheumatoid arthritis

George W. Reed, David H. Collier, Andrew S. Koenig, Katherine C. Saunders, Dimitrios A Pappas, Heather J. Litman, Joel M. Kremer, Sameer Kotak

SUPPLEMENTAL METHODS

Markov Chain

A Markov Chain or discrete-time Markov chain can be used to model state transitions. A Markov Chain is a Markov process with a discrete (countable) state space. The feature of a Markov Chain is that it is "memoryless" – the future state depends only on the current state. Let Y_n represent the state at time n. The probability of the state at the next time point (n+1) conditional on all prior states is reduced to conditional on the most recent state:

$$P(Y_{n+1}=j \mid Y_0=i_0, \ldots, Y_{n-1}=i_{n-1}, Y_n=i_n)=P(Y_{n+1}=j \mid Y_n=i_n)$$

Higher order Markov Chains where the future state depends on more than the most recent state can be used to expand the modeling framework. For example, second order Markov chains depend on the two most recent states:

$$P(Y_{n+1}=j \mid Y_n=i_n, Y_{n-1}=i_{n-1})$$

For the 2-state problem we can write down the transition probabilities and describe the transition matrix. Let 0 = remission/low state and 1 = moderate/severe state. For the ith patient we have the 4 possible transition probabilities:

$$\pi_{00} = P(Y_{ij} = 0 \mid Y_{ij-1} = 0)$$
$$\pi_{01} = P(Y_{ij} = 1 \mid Y_{ij-1} = 0)$$
$$\pi_{10} = P(Y_{ij} = 0 \mid Y_{ij-1} = 1)$$
$$\pi_{11} = P(Y_{ij} = 1 \mid Y_{ij-1} = 1)$$

Or the transition matrix:

$$\begin{array}{cc} & Y_{ij} \\ Y_{ij-1} & \begin{bmatrix} \pi_{00} & \pi_{01} \\ \pi_{10} & \pi_{11} \end{bmatrix} \end{array}$$

Note that

$$\pi_{00} + \pi_{01} = 1$$
$$\pi_{10} + \pi_{11} = 1$$

The 3-state problem expands the transition matrix since there are more transition choices.

$$\begin{array}{cc} & Y_{ij} \\ Y_{ij-1} & \begin{bmatrix} \pi_{00} & \pi_{01} & \pi_{02} \\ \pi_{10} & \pi_{11} & \pi_{12} \\ \pi_{20} & \pi_{21} & \pi_{22} \end{bmatrix} \end{array}$$

Using sequential patient data at each clinical visit we can estimate the transition probabilities in RA patients. The interest is in estimating the transition probabilities – ie, given a patient’s current state what are the probabilities of remaining in that state or moving – and estimating the association of covariates with those transition probabilities.

In order to model factors associated with the transition probabilities a logistic regression model is used for the 2-state problem. A model of the probability of transition to state 1 conditional on being in state 0 or state 1 at the prior visit can be written as to logit models:

$$\text{logit}\Pr(Y_{ij} = 1 \mid Y_{ij-1} = 0) = \beta_0^{'} x_{ij}$$
$$\text{logit}\Pr(Y_{ij} = 1 \mid Y_{ij-1} = 1) = \beta_1^{'} x_{ij}$$

Where x_{ij} are the characteristics of the patient.

The model can be written in a more compact form as:

$$\text{logit}\Pr(Y_{ij} = 1 \mid Y_{ij-1} = y_{ij-1}) = \beta_0^{'} x_{ij} + y_{ij-1}\alpha^{'} x_{ij}$$
$$\beta_1 = \beta_0 + \alpha$$

For the 3-state problem a multinomial logistic model would be used.

Table S1. Model with visit interval interaction

Covariate	OR (95% CI)
Prior state	
Low[a]	1
Moderate/severe	12.419 (10.941 to 14.097)
Interval group	
3 to <4 months[a]	1
4 to <5 months	1.032 (0.921 to 1.155)
5 to <6 months	1.042 (0.927 to 1.172)
6 to <7 months	1.146 (1.014 to 1.295)
7 to <8 months	1.281 (1.109 to 1.479)
8 to 9 months	1.146 (0.966 to 1.359)
Prior state and interval group interaction	
Moderate/severe and 4 to <5 months	0.914 (0.779 to 1.072)
Moderate/severe and 5 to <6 months	0.777 (0.658 to 0.917)
Moderate/severe and 6 to <7 months	0.691 (0.580 to 0.824)
Moderate/severe and 7 to <8 months	0.650 (0.527 to 0.802)
Moderate/severe and 8 to 9 months	0.801 (0.631 to 1.017)

[a]Baseline category.
OR, odds ratio; CI, confidence interval.

Table S2. Impact of visit interval on transition probabilities

Covariate	OR (95% CI)
Transition from low to moderate/severe disease	
3 to <4 months[a]	1
4 to <5 months	1.032 (0.921 to 1.155)
5 to <6 months	1.042 (0.927 to 1.172)
6 to <7 months	1.146 (1.014 to 1.295)
7 to <8 months	1.281 (1.109 to 1.479)
8 to 9 months	1.146 (0.966 to 1.359)
Transition from moderate/severe to moderate/severe disease	
3 to <4 months[a]	1
4 to <5 months	0.943 (0.846 to 1.051)
5 to <6 months	0.810 (0.722 to 0.908)
6 to <7 months	0.792 (0.701 to 0.895)
7 to <8 months	0.833 (0.717 to 0.968)
8 to 9 months	0.918 (0.778 to 1.082)

[a]Baseline category.
OR, odds ratio; CI, confidence interval.
